# Mis-Assembled “Segmental Duplications” in Two Versions of the *Bos taurus* Genome

**DOI:** 10.1371/journal.pone.0042680

**Published:** 2012-08-03

**Authors:** Aleksey V. Zimin, David R. Kelley, Michael Roberts, Guillaume Marçais, Steven L. Salzberg, James A. Yorke

**Affiliations:** 1 Institute for Physical Science and Technology, University of Maryland, College Park, Maryland, United States of America; 2 McKusick-Nathans Institute of Genetic Medicine, Johns Hopkins University, Baltimore, Maryland, United States of America; Georgia Institute of Technology, United States of America

## Abstract

We analyzed the whole genome sequence coverage in two versions of the *Bos taurus* genome and identified all regions longer than five kilobases (Kbp) that are duplicated within chromosomes with >99% sequence fidelity in both copies. We call these regions High Fidelity Duplications (HFDs). The two assemblies were Btau 4.2, produced by the Human Genome Sequencing Center at Baylor College of Medicine, and UMD Bos taurus 3.1 (UMD 3.1), produced by our group at the University of Maryland. We found that Btau 4.2 has a far greater number of HFDs, 3111 versus only 69 in UMD 3.1. Read coverage analysis shows that 39 million base pairs (Mbp) of sequence in HFDs in Btau 4.2 appear to be a result of a mis-assembly and therefore cannot be qualified as segmental duplications. UMD 3.1 has only 0.41 Mbp of sequence in HFDs that are due to a mis-assembly.

## Introduction

### Duplications: a challenge to genome assembly

Segmental duplications have been the focus of much of the biological analysis of the mammalian genomes [1], [2]. They play an important role in the evolution of many species, often providing a substrate for the development of new gene functions. Such duplications present a challenge to genome assembly, particularly when the duplications are recent and the copied sequences are near-identical. Assembly programs sometimes collapse nearby duplications into a single copy, or erroneously incorporate multiple copies of a unique sequence into the assembly. The creation of erroneous duplications can be caused by divergent regions in a diploid genome, in which the two haplotypes are sufficiently different that the assembler fails to merge them together. Identifying such mis-assemblies is critically important for downstream biological analysis.

### Two assemblies of the Bos taurus genome

In April 2009, two assemblies of the *Bos taurus* genome were published simultaneously: Btau 4.0 by the Baylor College of Medicine [3] and UMD Bos taurus 2.0 (UMD 2.0) by the University of Maryland [4]. These assemblies have since been updated, and the current versions are Btau 4.2, available from the sequencing center's website ftp://ftp.hgsc.bcm.tmc.edu/pub/data/Btaurus/fasta/Btau20080815/, and UMD Bos taurus 3.1 (UMD 3.1), available from Genbank as accession DAAA00000000.2. We note that Btau 4.2 has only minor differences from the published Btau 4.0 assembly; the primary update was the replacement of selected contigs by finished BAC sequences. In this paper we analyze the latest available versions of both assemblies.

## Results

### High Fidelity Duplications (HFDs) in the two assemblies of *Bos taurus* genome

One striking difference between the assemblies is the disparity in the number of large regions of sequence that are duplicated within the chromosomes with high fidelity between copies. We defined a High Fidelity Duplication (HFD) as any region >5 Kbp in length occurring in two copies in the assembly, such that the copies are >99% identical to each other and reside on the same chromosome. To find the HFDs we used the Nucmer software [5] to map each assembly to itself and looked for non-overlapping self matches longer than 5 kbp with at least 99% identity. Btau 4.2 has 3,111 HFDs, while UMD 3.1 has 69. More surprisingly, only 2 of these HFDs appear in both assemblies. The Btau 4.2 regions cover 83 Mbp of sequence, while the UMD 3.1 duplications cover 1.3 Mbp.

In this paper we present analysis that shows that almost all HFDs in the Btau 4.2 and some in UMD 3.1 are assembly artifacts and therefore should be ignored for biological analysis.

**Figure 1:**
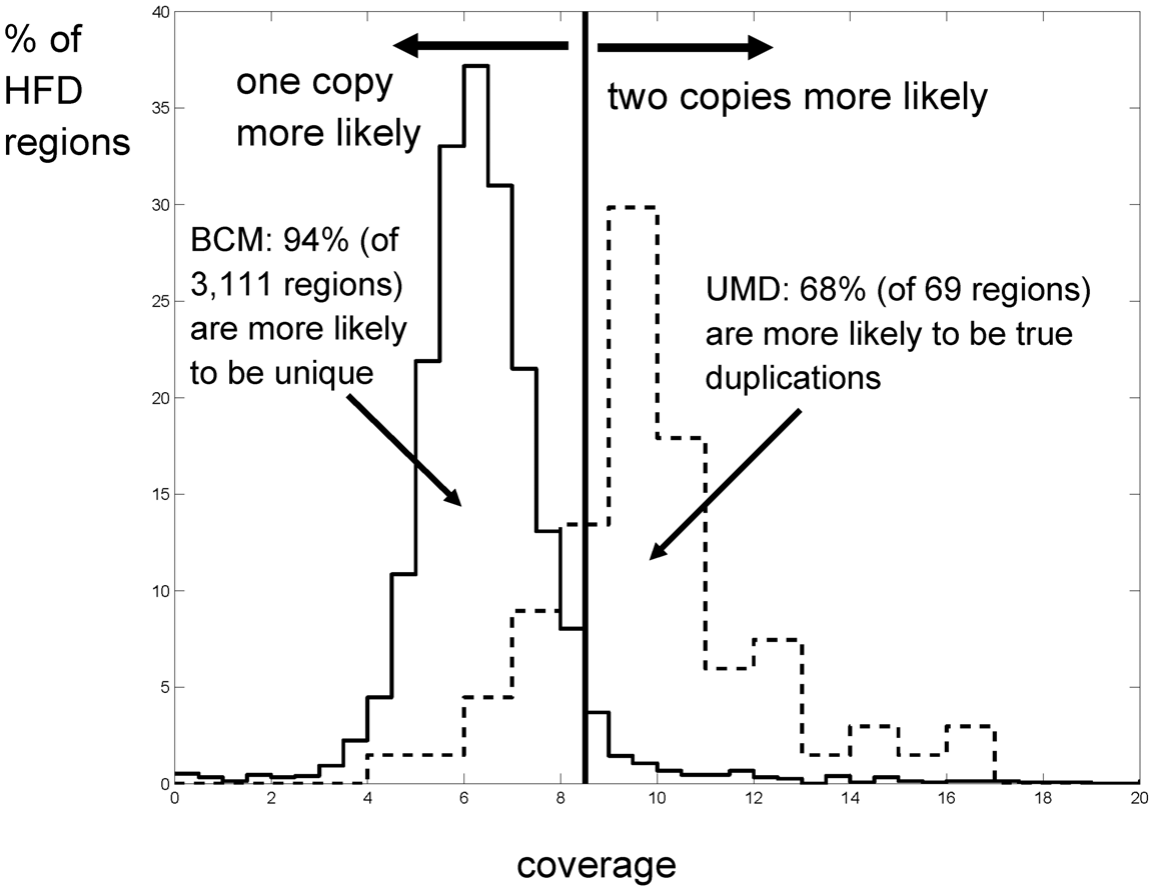
Histogram of the percentage of HFDs that belong to (i) set B2U1, duplicated in Btau 4.2 and single copy in UMD Bos taurus 3.1 (solid line), and (ii) set B1U2, single copy in Btau 4.2 and duplicated in UMD Bos taurus 3.1 (dashed line). The area under each curve integrates to 100%. The histograms were computed by mapping the WGS reads to both assemblies. The average WGS read coverage of the assemblies is 5.9. The solid vertical line is placed at 5.9/ln(2), the coverage at which it is equally likely that a region occurs in two copies versus one. 47 of the 69 regions (68%) in B1U2 are on the right hand side of the line and thus they are more likely to be true segmental duplications. 94% of the 3,111 HFDs in Btau 4.2 (set B2U1) are more likely to be unique in the genome and thus probably represent assembly errors in Btau 4.2.

Figure 1 shows the histograms of coverage for all HFDs in which the two assemblies disagree about copy number; i.e., at least one of the assemblies is incorrect. We created the set B1U2 containing the regions with exactly one copy in Btau 4.2 and two copies in the UMD 3.1 assembly; conversely, we created the set B2U1 containing the regions with two copies in Btau 4.2 and one copy in UMD 3.1. We show the distributions of read coverage for regions in B1U2 (dashed line) and B2U1 (solid line) as percentages of all regions. (Note that B2U1 is a much larger set, with 3,111 regions versus just 69 regions in B1U2.) Based on this WGS coverage statistic, 47 of the 69 regions (68%) in B1U2 are more likely to be true segmental duplications, suggesting that the UMD3.1 assembly is correct for these regions. In contrast, only 187 out of 3,111 regions (6%) in B2U1 appear to be true duplications, indicating that Btau 4.2 has a large number of erroneously duplicated sequences.

### Independent validation of false duplications in Btau 4.0

The BGSAC authors devote part of their paper to discussing the biological implications of the segmental duplications in their Btau 4.0 assembly. However, in the online supplement, they remark that many of their duplications are likely a product of mis-assembly: “A total of 1,860 pairwise alignments (>20 kbp, >94% identity) corresponding to 92.45 Mbp of apparent duplicated sequence in Btau 4.0 could not be substantiated by WSSD.” Note that these duplicated sequences were omitted from the main analysis, but they are still present in the 4.0 and 4.2 assemblies. Our analysis suggests that the problem is even more extensive since 84% of the regions that we analyzed are shorter than 20 Kb (but longer than 5 kb, see the definition of the HFD above), and therefore they had to be included in the main analysis.

These indications of erroneous duplications in the Btau 4.0 assembly are supported by a recent independent study by [6], which examined intra-chromosomal duplication patterns in the *Bos taurus* genome using fluorescent in situ hybridization (FISH). They compared Btau 4.0 and UMD 2.0 by analyzing 13 segments of the genome that were duplicated in only one of the assemblies. The FISH results were consistent with the UMD 2.0 assembly at 10 of 13 sites, while only 2 of 13 were consistent with Btau 4.0.

## Methods

### Evaluating the read coverage of duplicated regions in Btau 4.2 and UMD3.1

To determine which HFD regions are likely to be actually duplicated in the genome, we examined their whole-genome shotgun (WGS) read coverage. We aligned the WGS read sequences to each region that was a HFD in either assembly and calculated that region's read coverage, shown in Figure 1. We used Nucmer software to align the reads to the HFD sequences. We used a single copy of each HFD sequence because duplicate copies differed by less than 1% by definition. We accepted all matches with >94% identity over 90% of the read length. Mate pair information was not used. We then computed the coverage for each copy of the HFD as the total number of bases in the reads that match the HFD sequence divided by the length of the HFD. Next, we compared the individual read coverage of each HFD to the mean WGS read coverage over the entire genome, which was approximately 5.9×. In our analysis we assumed that WGS reads, which provided two thirds of the sequence data, were distributed nearly uniformly over the genome. Under this assumption, the coverage of the HFDs should have a Poisson distribution with a mean coverage of 5.9 in unique regions. If a sequence is truly duplicated in the genome and all reads are aligned to a single copy of that sequence, then the expected coverage would be twice the normal coverage, or about 11.8.

## Conclusions

Our analysis implies that that the BCM Btau 4.2 assembly contains at least 39 Mbp of intra-chromosomal duplicated sequence that appears to be single-copy in the genome. In contrast, UMD 3.1 has only 0.41 Mb that appear to be erroneously duplicated. A possible explanation for the excess duplications in Btau 4.2 can be found in the BAC-based assembly strategy used to construct it. The authors used a hybrid approach in which they first assembled bacterial artificial chromosomes (BACs) and then merged the BAC assemblies. Because the BACs were sequenced from either haplotype, when two overlapping BACs represented different haplotypes, sequence divergence might have prevented the assembly software from correctly merging them, and instead the BACs were assembled in adjacent, non-overlapping locations. This would create nearly identical duplicated sequences within chromosomes. We could not verify this conjecture because we do not have access to assembly sequences of individual BACs. Scientists analyzing the Btau 4.2 version of *Bos taurus* genome may need to gather additional, independent evidence before assuming that duplications in the assembly represent the true genome.
